# Disability experiences and quality of life in adolescent and young adult cancer survivors: a scoping review

**DOI:** 10.1007/s11764-025-01964-5

**Published:** 2026-01-16

**Authors:** Lindsey R. Vongthavaravat, Leeah M. Chang, Natasha Z. R. Steele, Amy Roder McArthur

**Affiliations:** 1 Rice University, Houston, TX U.S.A.; 2 Brown University, Providence, RI U.S.A.; 3 Stanford University School of Medicine, Palo Alto, CA U.S.A.; 4 Center for Education in Health Sciences, Feinberg School of Medicine, Northwestern University, Chicago, IL U.S.A.

**Keywords:** Disability, AYA, Americans with Disabilities Act, Cancer Survivorship, Legal Rights

## Abstract

**Purpose:**

This scoping literature review explored disability-related experiences among adolescent and young adult (AYA) cancer survivors [ages 15–39], including their knowledge of the Americans with Disabilities Act (ADA) and awareness of associated rights.

**Methods:**

Seven databases (PubMed, Academic Search Complete, MEDLINE, ERIC, SocINDEX with Full Text, Health Source: Nursing/Academic Edition, and APA PsychINFO) were searched for U.S. based literature (2010–2025) using relevant MeSH terms and boolean operators (e.g., “cancer surviv*” AND (“disab*” OR “impair*”) AND (“young” OR “adolescent” OR “AYA”). Included studies focused on employment-related discrimination, cancer-related disclosure, psychosocial outcomes, and survivors’ unmet disability-related needs. A narrative synthesis summarized key themes, with quantitative results tabulated and qualitative findings categorized.

**Results:**

A total of 715 articles were screened, with 21 studies included for analysis. These studies highlight key gaps in legal awareness and support for adolescent and young adult (AYA) cancer survivors. Three main themes were identified: (1) employment, education, and workplace barriers, (2) legal, policy, and system-level barriers, and (3) psychosocial and identity-related challenges.

**Conclusion:**

AYA cancer survivors encounter barriers in daily life and navigate changes to their social identity that could be described as disability based on existing legal and social definitions. Despite growing research capturing the experiences of AYA survivors, there remains limited research addressing survivorship in relation to disability.

**Implications for Cancer Survivors:**

Current survivorship literature lacks discussion of disability experiences of survivors and relevant disability support. Addressing this gap could better document the lived experiences of AYA survivors and design supportive services informed by disability law and policy.

**Supplementary Information:**

The online version contains supplementary material available at https://doi.org/10.1007/s11764-025-01964-5.

## Introduction

Cancer remains a significant public health challenge worldwide. Despite increased incidence in recent years, advancements in medical technologies and early detection methods have improved survival rates [1]. Adolescents and young adult (AYAs) cancer survivors — defined as individuals who are diagnosed with cancer for the first time between the ages of 15 and 39 — represent a unique and rapidly growing subgroup within cancer survivors, with over 2.1 million AYA survivors in the United States alone as of January 2020 [2]. By 2040, the number of U.S. cancer survivors is projected to be 26 million [3].

While achieving post-treatment survivorship is a significant milestone, cancer survivors face many long-term and late effects of cancer, including treatment-related physical symptoms, psychosocial struggles, and difficulties with reintegration into educational, professional, and social settings [4, 5]. For AYAs, these challenges can be particularly impactful, as diagnosis occurs during a critical developmental window characterized by increasing independence, early-stage careers, and formation of romantic and professional relationships—milestones that significantly shape long-term quality of life and future trajectories.

The impact of cancer on AYA survivors is distinct as their biological, medical, and psychosocial experiences differ from those of pediatric and geriatric cancer patient populations. For example, AYA cancer survivors who undergo high-dose chemotherapy or radiation therapy have an increased risk of developing chronic health issues, such as hearing loss and heart failure [6, 7]. Additionally, this population experiences higher rates of psychological distress, largely due to financial burden, frequent healthcare utilization, and cognitive dysfunction, which can adversely affect employment and overall quality of life [7, 8]. Given the distinctive period of life of AYA cancer survivors, disruptions in this period of life include negative net worth, financial decline over time, and difficulties in returning to education [9]. Concerns about job loss or reassignment to a lower position can pressure survivors to remain employed and discourage cancer-related disclosure for workplace accommodations [10].

Many of the experiences of AYA cancer survivors fall under existing definitions of disability. The Americans with Disabilities Act (ADA), the federal civil rights law that protects people with disabilities from discrimination and ensures equal opportunity, defines a person with a disability as someone who “has a physical or mental impairment that substantially limits one or more major life activities, has a history or record of such an impairment, or is perceived by others as having such an impairment” [11]. The ADA Amendments Act of 2008 broadened the definition of disability, including impairments that could be caused by cancer [12]. Under the ADA, people with a history of cancer are protected from discrimination in the workforce, state and local government services, public transit, businesses that are open to the public, and telecommunications. Although protected from discrimination by the ADA, about one-quarter of cancer survivors report experiences of workplace discrimination, with younger cancer survivors more likely to allege employment discrimination [10, 13, 14]. Additionally, many survivors are unaware of their legal rights, limiting their access to reasonable accommodations and other protections that can support continued employment and participation in their community [13, 15, 16]

Despite a growing amount of research on adolescent and young adult (AYA) cancer survivors, there is limited evidence documenting how cancer-related disability is understood and how survivors interact with disability-related frameworks. For AYA cancer survivors, impairments—such as physical, cognitive, and psychosocial symptoms—are closely tied to lower health-related quality of life [17]. Given the unique developmental stage of AYAs and the legal protections under the Americans with Disabilities Act (ADA), this scoping review aims to analyze existing literature, identify gaps in this research area, and investigate how AYA survivors understand and navigate disability, access disability-specific legal protections and accommodations, and manage factors that impact psychosocial integration.

## Methods

### Study design

**Fig. 1:**
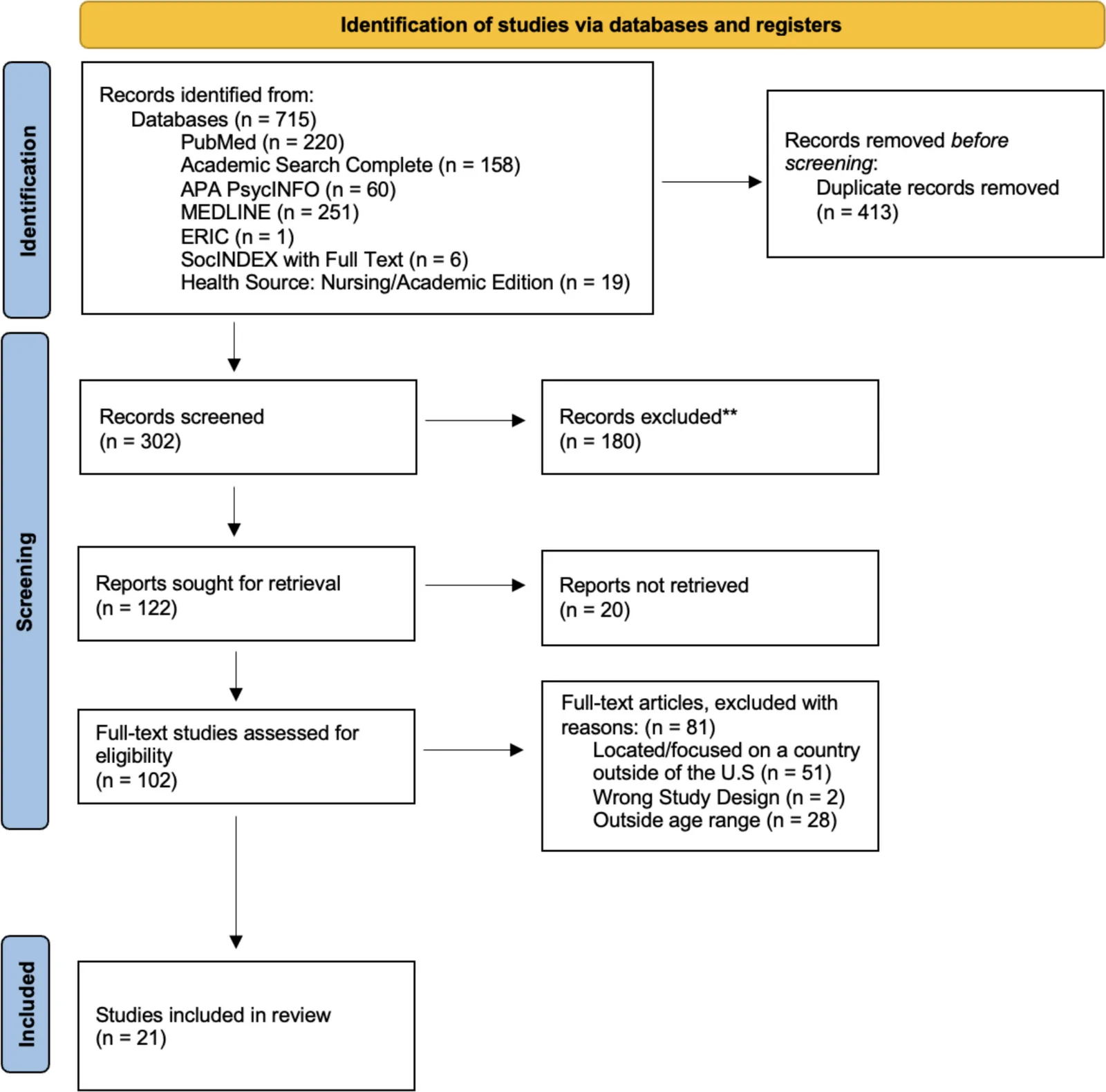
PRISMA 2020 Flow diagram illustrating the identification, screening, eligibility, and inclusion process for studies in this scoping review

This study follows a scoping review methodology to explore the relationship between adolescent and young adult (AYA) cancer survivors and disability as defined by the Americans with Disabilities Act (ADA). This review was conducted in compliance with the Preferred Reporting Items for Systematic Reviews and Meta-Analyses (PRISMA) guidelines. The screening and selection process is depicted in Fig. 1.

### Search strategy

A comprehensive literature search was conducted using the following electronic databases: PubMed (United States National Library of Medicine); Academic Search Complete, MEDLINE (Medical Literature Analysis and Retrieval System Online); ERIC (Education Resources Information Center); SocINDEX with Full Text; Health Source: Nursing/Academic Edition; APA PsycINFO (American Psychological Association). The search aimed to identify relevant peer-reviewed articles that address legal, medical, and social perspectives of disability in AYA cancer survivors.

Boolean operators and truncation were structured around three core conceptual domains.

1. Cancer Survivorship (“cancer surviv*”)
2. Disability-related concepts (e.g., “disab*”, “impair*”, and “Americans with Disabilities Act”)
3. Adolescents and young adults (e.g., “young”, “youth”, “adolescent”, “young adult”, “Adolescent and Young Adult”, “child*”, “teen*”, “AYA”, and “pedi*”)

Broad search terms such as cancer, rehabilitation, and limitations were excluded to narrow article results. To ensure comprehensiveness, the reference lists of eligible articles were also manually screened for additional relevant publications.

### Eligibility criteria

Studies and relevant papers were filtered and included if they met the following criteria: research focused on adolescent and young adult (AYA) cancer survivors (ages 15–39), or if they included this age group within a broader population and addressed themes directly relevant to disability and survivorship. Studies that included but did not exclusively focus on the AYA age group were classified as ‘Partial AYA’ and were retained for their conceptual relevance. Studies were also filtered to address themes related to cancer-related disclosure, the impact on work and education, knowledge of ADA rights, and psychosocial and emotional well-being. Both qualitative and quantitative studies, as well as systematic reviews and meta-analyses published between 2010 and 2025, were considered. Articles had to be peer-reviewed, based in the United States, and published in English.

Studies were excluded if they focused primarily on pediatric or geriatric populations, did not address the specified themes, were non-peer-reviewed, or were published before 2010. Non-English articles, studies based outside of the United States, and those without full-text availability were also excluded. In terms of relevance, studies were excluded if they primarily focused on other types of chronic illnesses, were editorial pieces, or did not provide data relevant to AYA cancer survivorship and disability. This eligibility filtering process is presented below in Table 1.

**Table 1:** Screening Exclusion and Inclusion Criteria

Criterion	Inclusion	Exclusion
Population	Studies involving adolescent and young adult (AYA) cancer survivors aged 15–39	Studies focused on pediatric, geriatric, or non-AYA cancer populations
Study Focus	Studies examining psychosocial challenges and legal protections related to employment discrimination under the ADA	Studies addressing only biological, medical, or clinical aspects or lacking relevance to ADA and cancer survivorship
Types of Studies	Peer-reviewed qualitative and quantitative studies, including systematic reviews and meta-analyses	Non-peer-reviewed articles, editorials, abstracts without full-text, and studies with small or non-generalizable samples
Publication Date	Published between 2010–2025	Published prior to 2010
Location	Studies conducted in the United States	Studies conducted outside the United States
Language	Articles written in English	Articles written in languages other than English

### Data extraction and management

After duplicate removal, all titles and abstracts were screened independently by the first and second authors for relevance to the inclusion criteria. Full texts of potentially eligible studies were retrieved and assessed for eligibility. Data extraction focused on study characteristics (e.g., sample size, population demographics, study design), key findings related to disability awareness, barriers to accommodations, and the quality of life of AYA cancer survivors. A standardized data-charting form was created to facilitate variable extraction and was piloted on a subset of studies to ensure consistency and clarity. Both reviewers independently charted data for all included studies, with discrepancies resolved through discussion.

### Data synthesis

A narrative synthesis approach was employed to summarize findings across the included studies. Data collected were organized thematically into categories reflecting legal awareness and barriers, employment and education barriers, and psychosocial and identity-related experiences. Where applicable, quantitative data were tabulated, and qualitative findings were analyzed for emerging themes.

### Ethical considerations

This review did not involve direct human subjects or the collection of primary data. As such, ethical approval from an institutional review board was not required.

## Results

### Study selection

A total of 715 records were identified through initial database searches. Following the removal of duplicates, 302 articles were selected for screening. After title/abstract screening, 122 articles were chosen for full-text retrieval. Following the application of inclusion and exclusion criteria, 21 studies were included in the final review. The full screening process is outlined in the PRISMA flow diagram (Fig. 1).

### Study characteristics

The 21 included studies were published between 2010 and 2025. Study designs varied and included qualitative studies (n = 4), retrospective cohort studies (n = 1), retrospective qualitative studies (n = 1), prospective observational cohort studies (n = 1), cross-sectional observational studies (n = 6), descriptive studies (n = 1), quantitative (n = 2), qualitative case studies (n = 1), narrative review (n = 2), thematic synthesis (n = 1), and quasi-experimental (n = 1). All studies were conducted in the United States and published in peer-reviewed journals. Many studies were focused on the medical, psychological, and social science disciplines.

Among the 21 studies, 17 focused on adolescent and young adult (AYA) cancer survivors defined as ages 15–39, while the remaining 4 studies included broader adult and pediatric cancer populations. These 4 studies were retained due to their strong thematic relevance to disability, ADA awareness, and close applicability to AYA populations.

In Table 2, the key characteristics of the included studies are summarized by author(s), publication year, study design, patient population, AYA focus, themes, and key findings.

**Table 2:** Summary of Studies Included in Scoping Review of Disability and the Cancer Experience in AYAs

Author(s), Year, Reference Number	Study Design	Population (n, age)	AYA Focus	Themes	Study Aim	Key Findings
Strauser et al., 2010 [20]	Retrospective Cohort Analysis	368 young adult survivors (18–25 years)	Yes	Employment, Vocational Services	Examine how vocational support services influence employment outcomes among young cancer survivors who were unemployed prior to receiving such services	Job support and search assistance services improved survivors’ employment odds. Cash or Medical Benefits (SSA) decreased the likelihood of employment. 51.6% of survivors receiving vocational rehabilitation achieved employment
Parsons et al., 2012 [4]	Prospective Observational Cohort	463 survivors (15–39 years)	Yes	Return to Work/Education	Determine the impact of cancer on AYA cancer survivors’ ability to participate in work and education	72% of survivors returned to full-time work or school. Lack of insurance and quitting school/work were associated with lower return rates. More than 50% of survivors reported post-diagnosis problems with work/education
Yanez et al., 2013 [33]	Cross-Sectional Observational	335 young adult cancer survivors (mean age = 31.8)	Yes	Distress, Quality of Life	Determine the prevalence of distress and contributing factors among AYA cancer survivors (ages 18–39)	Psychosocial factors such as distress are critical factors contributing to minimal disclosure, particularly in terms of education/work interruption and continuation. Clinically elevated distress is linked to cancer-related education/work disruption. Survivors 13–60 months post-treatment with work interruption have significantly higher distress (p < 0.05) than those 0–12 months post-treatment
Barnett et al., 2014 [21]	Qualitative (grounded theory)	26 survivors (16–24)	Yes	Cancer-Related Disclosure, Psychosocial Identity	Explore how AYA cancer survivors diagnosed in adolescence choose to disclose their cancer history	Cancer-related disclosure, including both willingness and patterns, is shaped by decision-making, methods of disclosure, and perceptions of others’ responses. Additionally, the length of time post-treatment and perception of anticipated response play a role in one’s decision to disclose. Females represented frequent disclosure, and the “it depends” theme dominated
Bashore & Breyer, 2017 [18]	Descriptive	50 survivors (12–18)	Partial	Education, Career	Investigate the educational and vocational outcomes of young adults who survived childhood cancer	Physical impairments associated with central nervous system tumor and radiation. Themes included disrupted career goals, peer difficulties, and resilience outlooks
Psihogios et al., 2018 [28]	Quasi-experimental (pre-post educational intervention)	40 college health staff (24 institutions), 18 health center providers	Yes (Focused on YAS = Young Adult Survivors)	Awareness of survivorship resources, provider knowledge	Evaluate the baseline knowledge of college providers on health risks of YAS and relevant survivorship care guidelines, and to examine whether an educational in-service could effectively enhance their understanding within a large university health center	Providers lacked a baseline understanding of survivorship guidelines and services. Post-intervention caused improvements in knowledge, comfort with follow-up care, and resource awareness
Benedict et al., 2018 [35]	Retrospective qualitative (grounded theory)	46 survivors (mean age = 32)	Yes	Fertility, Financial, and emotional burden disrupted life trajectory	Examine the experiences and financial challenges faced by survivors who pursued family building through assisted reproductive technology (ART) or adoption	Survivors faced emotional distress, financial barriers to ART/adoption, and disrupted life planning. Family-building challenges caused extreme financial stress and role-related guilt
Crom et al., 2018 [23]	Cross-sectional observational	289 survivors of childhood cancer (50 aged 18–29)	Partial	Workplace discrimination, turnover intention	Explore survivors’ workplace experiences and their intentions to leave their jobs, as well as identify factors associated with turnover intention	30% reported high turnover intention, or the likelihood of leaving a job within a certain period. A higher intent to leave was linked with cranial radiation therapy (CRT), workplace discrimination, and low job commitment. Job satisfaction mediated the effects of informal satisfaction. Clinical staff are well-positioned to provide legal assistance regarding survivors’ rights under the ADA before they re-enter the workforce
Ketterl et al., 2019 [24]	Cross-sectional observational	872 survivors (18–39), 1–5 years post-diagnosis	Yes	Cancer's lasting effects, Employment, Financial Burden	Assess how cancer-directed treatment affects young adult survivors’ (ages 18–39) physical and mental ability to perform work tasks, their need to modify employment or take paid/unpaid leave, and the financial toxicity experienced by survivors and their families	Cancer treatment is associated with increased physical and mental impairment in work tasks. Unpaid time off and large borrowing were reported. Late effects were varied by diagnosis and treatment (e.g., radiation, chemo, surgery)
Scardaville et al., 2019 [16]	Cross-sectional survey	203 young adult survivors (ages 18–39)	Yes	Legal Policies, Employment	Determine the knowledge of young adult cancer survivors of legal and programmatic supports and preferred methods to receive information about employment-related issues	57% of respondents knew of ADA, but most were unaware that cancer was covered. ~ 80% were unaware of other federal protections; preferred delivery methods were in-person training and fact sheets. Sociodemographic factors influenced awareness
Stone et al., 2019 [26]	Qualitative (grounded theory)	343 survivors from the Los Angeles County Cancer Surveillance Program (ages 15–39)	Yes	Workplace interactions, Occupational health support, Disclosure	Characterize the work experiences of long-term young adult cancer survivors and examine their interactions with occupational and environmental health professionals (OEHPs) in the workplace	Survivors’ work ability was shaped by disclosure, perceived support, and gatekeeping by occupational health professionals. Survivors’ knowledge and willingness to seek support determined the level of access to services
Naughton et al., 2020 [30]	Quantitative, cross-sectional	708 women ≤ 45 years old with stage I–III breast cancer	Yes (≤ 45 year old)	Insurance concerns; unemployment/disability status	Assess the prevalence and correlation of job/insurance problems during the first 18 months post-diagnosis for AYA breast cancer survivors in the U.S	40% of survivors reported job-related issues (e.g., fear of job change, firing, demotion). 27% had insurance issues, and the authors recommended addressing work/insurance early in care. Risk factors: obesity, lower income, not on hormone therapy, and larger tumor size. Current and ongoing modifications to laws in the healthcare space are not necessarily helping ease workplace and/or insurance concerns of young cancer survivors
Rabin et al., 2020 [25]	Quantitative, cross-sectional survey	120 cancer survivors (diagnosed ages 15–39, surveyed ages 19–45)	Yes (15–39 years at diagnosis)	Cancer-related disclosure, workplace/education support	Determine the factors that affect cancer-related self-disclosure in work and school settings, and strategies used for self-disclosure	Survivors were significantly less likely to self-disclose cancer history at work/school than to friends. Positive reactions to previous disclosures increased the likelihood of future disclosure. Survivors used strategies such as humor and gauging reactions. Authors call for more support environments in school/work
Strauser et al., 2020 [14]	Qualitative case study using the Work Experience Survey (WES)	4 young adult brain cancer survivors (employed) [ages 26–32]	Yes	Job accessibility, ADA accommodations	Determine the effectiveness of the Work Experience Survey (WES), an on-the-job needs assessment tool, to assess barriers to employment and career development for young adult brain cancer survivors	Participants reported barriers related to accessibility, job mastery, and satisfaction due to cognitive/mobility impairments. WES identified job accommodation needs, which included Job Accommodation Network consultants’ suggestions. Study supports the use of WES for planning job modifications
Blanck, 2022 [10]	Thematic special section synthesizing research and commentary (includes 5 articles)	Cancer survivors and people with disabilities, with relevance to AYAs	Yes (addresses survivors generally, including AYAS	ADA, employment accommodations, disclosure	Provide analysis of how the Americans with Disabilities Act (ADA) affects the employment experiences of AYA cancer survivors, noting persistent discrimination in application of protections for marginalized demographics	ADA laws enforce structural change, but discrimination persists. Cancer survivorship cases under the ADA tend to be seen on a case-by-case basis to determine the extent of barriers encountered. The author highlights the need for disability-inclusive employment research, especially for marginalized subgroups in terms of race, ethnicity, ableism, gender identity, and/or sexual orientation. COVID-19 exacerbated inequities in employment for survivors. This study spotlights the introduction of two new national RRTCs via relatively recent ADA case law, focused on disability-inclusive employment
Ghazal et al., 2022 [22]	Cross-sectional online survey	40 young adult hematologic cancer survivors (ages 20–39) within 5 years post-treatment	Yes	Quality of life, Work	Examine how individual factors and microsystems (workplace) characteristics relate to quality of life (QOL) among young adult hematologic cancer survivors diagnosed between ages 20 and 39	Poorer quality of life is associated with depressive symptoms, fatigue, impaired cognitive function, poor work ability, and worse financial health. Study highlights needs for multilevel work-related interventions and recommends broader, more diverse future samples
Magasi et al., 2022 [31]	Qualitative exploration	30 adult cancer survivors (breast, head and neck, sarcoma)	Indirectly (not specifically AYA-focused)	Disability Identity	Investigate the manifestation of long-term disability effects among cancer survivors, particularly in how they contribute to hindering or forming a disability identity	Survivors reported a range of disabling long-term effects. Reactions to disability identity varied (rejection, othering, acknowledgement, affirmation). Findings underscore the need for anti-ableist care and open discussions of disability
Carey et al., 2023 [19]	Narrative synthesis (literature review)	Students with chronic/complex medical conditions, focusing on childhood cancer survivors	Partial (childhood cancer survivor in school years)	Assistive technology	Provide information about assisted technology (AT) and its benefits for cancer survivors, how to gain access to such resources, and methods to remove possible barriers to AT	Childhood cancer survivors eligible for special education often lack access to needed assistive technologies. Schools should adopt research-based strategies to improve assessment, acquisition, and use to support students with health-related needs
Crowder et al., 2023 [34]	Qualitative	24 young adult cancer survivors (ages 18–39 years old)	Yes	Psychosocial functioning, Quality of life	Explore the post-treatment lived experiences of AYAs as well as quality of life among AYA cancer survivors, specifically regarding psychosocial challenges, coping mechanisms, and health behaviors	Young adult survivors experienced varying psychosocial concerns and coping strategies depending on the time since treatment. Key themes: symptoms, fear of recurrence, job/fertility concerns, and emphasis on post-treatment healthy behaviors
McGrady et al., 2024 [32]	Narrative review	AYA cancer survivors	Yes	Cancer-related cognitive impairment (CRCI), employment challenges, unmet psychological & health needs	Review psychological outcomes and challenges among AYA cancer survivors, spanning emotional, social, and health functioning domains	AYA survivors face a high risk of CRCI, emotional distress, and poor social/occupational outcomes. Many survivors fail to meet behavioral health guidelines. Review suggests interventions should address developmental needs and system-level supports
Robert et al., 2024 [29]	Cross-sectional	214 survivors (18–39 years)	Yes	Financial distress, workplace	Examine factors contributing to financial distress among AYA cancer survivors, namely material resources, health, and workplace-related influences, as a means of informing future strategies for provider guidance and resource development	Participants’ financial distress was attributed to material resources, physical and psychological health, and workplace variables. Study suggests that providers ought to discuss finances with survivors and provide necessary resources to maximize quality of life post-treatment

## Thematic findings

This scoping review identified three main themes: (1) employment, education, and workplace barriers, (2) legal, policy, and system-level barriers, and (3) identity and psychosocial adjustment.

## Employment, education, and workplace barriers

AYA cancer survivors in the U.S. face significant barriers when engaging in school or work settings. These barriers result from both individual factors, such as ongoing symptoms, and external environmental factors like social stigma, insurance-related barriers, and employment discrimination.

A recurring theme across the literature highlights the impact of treatment-related latent effects such as chronic fatigue, cognitive impairment, and physical limitations that often interfere with survivors’ ability to consistently engage in professional settings and activities [4, 18–21]. These persistent health-related limitations can not only impact productivity but can also undermine survivors’ confidence, increasing fear of underperformance and contributing to a poorer quality of life at the individual level [22]. Survivors in the included studies encountered disruptions to their career trajectories related to the presence of physical and mental impairments following a cancer diagnosis. These disruptions contributed to employment disparities for AYA cancer survivors, including longer periods of unpaid leave, career interruptions, and higher turnover intentions in the workplace [23, 24]. Survivors also face various social and structural barriers that exacerbate educational and employment challenges. These include concerns about being perceived as less capable by employers, peers, coworkers, or educators. Concerns about being perceived differently by others contribute to a reluctance for cancer survivors to disclose a history of cancer or identify publicly as a “survivor.” The difficulty in attaining high job satisfaction, or the need for basic modifications, only adds to the reluctance to disclose such information [14]. As found in the literature, disclosure decisions are shaped by several factors, including but not limited to workplace culture, anticipated stigma, and survivors’ own self [21, 25]. The decision to disclose a cancer diagnosis depends on a number of factors, including workplace or school stigma, to negative self-perceptions as a result of psychosocial challenges [21]. Additionally, reactions to previous disclosures tend to influence the likelihood of future disclosures, further perpetuating the stigma surrounding cancer survivorship [25]. For those who decide to return to school or work, discrimination can remain prevalent, with a high turnover intention observed among survivors due to workplace bias [23]. As noted by Stone et al. (2019), survivors' comfort and willingness to disclose were found to be linked with their work ability, which was directly correlated with their perceived level of support and access to specific support services in the workplace [26]. Because access to supportive services in the workplace is typically initiated by the employee, increased hesitancy for disclosure can limit AYA survivors’ access to resources, such as job support or search assistance services, which have been found to streamline retention and reintegration in educational or work environments [27].

These employment disruptions have potential long-term effects on lifetime income and career trajectory for survivors. In the educational context, institutional support for AYAs was critical in facilitating psychosocial adaptation following a cancer diagnosis. Psihogios et al. (2018) found that college health providers and disability administrators often lacked adequate training in cancer survivorship, contributing to fragmented care and a lack of early intervention and support. College health providers, college administration, and disability staff mutually agreed that there is a need to facilitate and distribute effective education regarding the medical and psychosocial needs of cancer survivors [28]. Educational institutions, such as universities, provide a unique opportunity to support survivors’ reintegration into the educational environment.

## Legal, policy, and system-level barriers

The Americans with Disabilities Act (ADA) is a federal civil rights law that offers explicit legal protections for individuals with disabilities, including those diagnosed with cancer whose cancer or treatment-related late effects limit one or major life activities [10].

AYA survivors demonstrated the need for further knowledge of how federal legislation, such as the Family and Medical Leave Act (FMLA) or the Rehabilitation Act, may be relevant to managing survivorship; [16]. Despite knowledge of relevant law, such as the ADA, Blanck (2022) described the difficulties encountered by cancer survivors when attempting to access ADA protections. Although the ADA has the potential to protect survivors from workplace discrimination based on their history of cancer, there is a general lack of knowledge of the ADA among cancer survivors and even employers that inhibits its full effectiveness as a landmark civil rights law [23]. Another study by Robert et al. (2024) identifies gaps in policy implementation, noting that high financial distress and inconsistent institutional procedures often prevent young adult survivors from accessing accommodations. As such, there is a strong need to promote concurrent labor and healthcare policy reform because even when survivors are aware of their rights, the financial and general burden of cancer can often limit their ability to effectively use the protections that are available to them [29].

Other authors highlighted the need to proactively support cancer survivors in their engagement in employment or education. Crom et al. (2018) highlights the importance of workplace policies that proactively support survivors in employment through employee advocacy and provide realistic solutions, rather than relying solely on legal protections to address employment discrimination. Further, this study noted that there is an increased likelihood that cancer survivors who do file discrimination claims under the ADA will be validated compared to other individuals with other disabilities who file workplace discrimination lawsuits [23]. Naughton et al. (2020) detailed the effects that insurance and employment policy concerns had on a participant pool of young breast cancer survivors. One concern highlighted was how possible changes to and general uncertainty regarding the extent of certain provisions under the Affordable Care Act (ACA) could pose more insurance, employment, and subsequent financial challenges for young cancer survivors, given the possibility of certain Americans losing coverage [30].

## Identity and psychosocial adjustment

Though research indicates the number of downstream psychosocial effects that influence AYA survivors’ sense of self and trajectory of life, few explicitly describe these changes in relation to disability.

Magasi et al. (2022) described how cancer survivors responded to the possibility of adopting a disability identity in survivorship. This study revealed a variety of perspectives among cancer survivors towards their disability status, ranging from rejection to othering to affirmation. The survivors who acknowledged or affirmed their disability identity in this study had often been previously exposed to disability rights or were experienced in navigating the process of accessing disability services and accommodations. Restructuring their personal identity following cancer was shaped by environmental and interpersonal factors, including their peers’ reaction to disclosure, access to accommodations, and personal sense of competence [31]. This was the only study in our review that explored disability as an identity category available to cancer survivors. While identity reconstruction is important to understand how survivors view themselves in relation to disability, their capacity and willingness to do so are often shaped by the psychosocial context of survivorship.

AYA cancer survivors face persistent psychosocial challenges that can shape how they engage in their daily lives throughout survivorship. AYAs often experience significant levels of emotional distress in comparison to the general population [32]. Distress was not confined to a singular point in survivorship; rather, it varied by diagnosis, intensity of treatment, and the time since treatment. Distress levels were the highest among survivors in the 13 to 24-month post-treatment period, a period associated with the transition back into daily life and responsibilities [33]. Elevated distress was significantly associated with cancer-related disruptions in education and employment as survivors encountered economic instability and struggled to regain their footing in school and the workforce. AYA cancer survivors experience a wide range of psychosocial challenges, including anxiety, reduced self-esteem, body image issues, and concerns related to fertility and appearance [34]. Importantly, the nature of these challenges changes over time. Survivors who were in the year after treatment experienced effects such as fatigue, hair loss, and limitations with their range of motion. In comparison, survivors who were three years or more post-treatment experienced symptoms and concerns impacting their quality of life, largely because of lingering treatment effects, such as fatigue, hormonal changes, and feelings of life stress. These differences highlight the evolving nature of survivorship, emphasizing the chronic but developing nature of post-cancer effects. The lasting physical and emotional effects of cancer and its contribution to cancer-related distress are closely associated with disability, not only by imposing bodily limitations but by impacting survivors’ capacity to fully participate in work, education, relationships, and other meaningful aspects of daily life [34]. Distress, in this sense, can be considered more than an emotional response and is seen as part of a broader disabling context that shapes how AYA survivors reconstruct identity and navigate the social world after cancer.

Family-building after cancer constitutes an additional psychosocial challenge for AYA survivors and a contributor to increased emotional distress. Many survivors faced compounding financial pressures stemming from cancer-related financial toxicity, financial stress caused by family-building, and expenses associated with school and career. Survivors faced structural barriers in accessing assisted reproductive technologies (ART) and adoption services, which intensified emotional distress and contributed to the perceived gap between themselves and peers who were progressing along conventional life milestones [35].

These findings highlight a small percentage of the many personal and social changes that AYA cancer survivors navigate as they engage in daily life following active treatment.

## Discussion

This scoping review explores how adolescent and young adult (AYA) cancer survivors experience disability within the US, including perspectives related to legal protections under the Americans with Disabilities Act (ADA) and psychosocial identity. Across the 21 studies included in this review, key themes emerged highlighting barriers to cancer-related disclosure, psychosocial distress, vocational and educational disruption, and limited legal and financial support.

Of note, only one article in our review characterized disability as a potential identity category relevant to survivorship, whereas the majority of the other literature described cancer-related disability in relation to the presence of lasting symptoms. The factors encompassing a survivor’s identity were seen to be influenced by their community experiences, internal expectations, and societal stigma [31]. Additionally, disability is defined in various ways and may be understood by cancer survivors in complex ways. Legally, the Americans with Disabilities Act (ADA) defines disability as “a physical or mental impairment that substantially limits one or more major life activities,” protecting those who currently have cancer or have had it in the past. These protections entitle survivors to engage in public life without discrimination. This can include access to reasonable accommodations or other supports that safeguard them against employment and educational exclusion. In contrast, disability is defined by the World Health Organization (WHO) as a result of the interaction between one’s health conditions and their personal and environmental factors, reflecting a biopsychosocial perspective that integrates aspects of both the medical and social models of disability [36].

Whether or not a cancer survivor identifies as disabled, they still may be protected by the ADA or have access to disability related support. Without adequate knowledge of their legal protections, survivors may be unsure how to navigate and define their experience with disability, possibly perpetuating unmet needs across educational, occupational, and psychosocial settings.

To access disability related rights or support, AYA survivors must first disclose information related to their cancer history or lasting symptoms, based on the current structure of disability support within employment and education spaces. Disclosure of a cancer history or post-treatment impairment is shaped not only by individual readiness but also by institutional norms and societal perceptions. Many survivors navigate long-term symptoms such as fatigue and cognitive impairments that may appear invisible from the outside perspective, but significantly impact their daily functioning, further requiring them to disclose their access needs in various settings [5, 32]. Reactions to a survivor’s previous disclosure attempts can also discourage future disclosures, perpetuate a cycle of silence, and prevent survivor self-advocacy. Future research should examine factors that encourage or hinder AYA cancer survivors from disclosing their diagnosis history, access needs, and lasting symptoms to understand how to support this population in realizing the rights granted to them under the ADA and other relevant disability laws.

This scoping review includes 21 studies; this number reflects the limited amount of research assessing AYAs’ experiences in relation to disability. Within this review, several articles included populations outside the AYA age range, further emphasizing the limited amount of research in this field. These findings demonstrate a greater need for more targeted research in this area, especially in the context of the AYA population, given the importance of this life stage in shaping the lifetime trajectory of individuals. Although included studies in this review were required to be conducted in the United States, most of the research centered on understanding the relationship between AYA survivors and disability was conducted at foreign institutions, emphasizing the gap in research and knowledge of AYAs’ experiences within the societal and legal framework of the United States. Though existing research suggests that cancer survivors may be unaware of their rights under the ADA, it is not clear what the best mechanism is to improve this awareness or translate this knowledge into meaningful changes in employment, education engagement, or other aspects of daily life. Additionally, findings from the studies in this review raise important questions about how, if at all, disability is addressed in clinical settings. Current guidance from the National Cancer Institute’s Standards for Survivorship care recommends that survivors be screened for employment-related concerns and provided with appropriate referrals that could assist in the management of cancer-related disability, such as rehabilitation services; however, health systems are responsible for determining implementation details for these recommendations [37].

Though non-U.S. conducted studies were excluded from this review, it is still valuable to consider current international findings that may help inform future relevant research in the United States. For instance, common themes across countries like Japan, Great Britain, Norway, and Mexico indicate that AYA cancer survivors often experience inconsistencies when it comes to application of anti-discrimination or disability rights legislation as well as variabilities in compliance among employers [38, 39]. While these legal frameworks are comparable to the ADA in terms of providing reasonable accommodations as warranted, the general framework does differ, underscoring the need for further research as it pertains to how AYAs in the U.S. navigate ADA protections in American employment settings [39].

This study has limitations that should be acknowledged. Some studies examined general cancer survivor populations rather than focusing exclusively on AYAs. While these studies provide critical context on disability-related issues, their findings may not fully capture the age-specific experiences of AYA survivors. This reflects a broader gap in the literature and highlights the need for more research focused specifically on the intersection of disability and survivorship in the AYA population. Because formal risk-of-bias assessment is not required for scoping reviews, no standardized critical appraisal of included studies was applied in this review. However, general indicators of study quality, such as relevance to the research question and study population, methodological transparency, and rigor of study design and analysis, were considered during data extraction and synthesis. Therefore, variation in risk of bias across included studies may have influenced the strength and comparability of available evidence. Methodological limitations, such as database indexing, keyword variability, and the exclusion of non-U.S. studies, may have further constrained the breadth of the included literature. Additionally, the various definitions of disability presented in the literature may make it challenging to construct a comprehensive and cohesive understanding of the research in this area.

This scoping review sheds light on the complex relationship between AYA cancer survivorship and disability within the United States. Although many AYA survivors experience long-term physical, cognitive, and psychosocial impairments that qualify survivors for legal protections under the ADA, few self-identify as disabled. Factors such as internal and environmental stigma and limited awareness of legal protections contribute to the nuanced experience of cancer survivors with support services among educational and professional institutions. The findings of this scoping review reveal a critical need to integrate disability education into survivorship care, promote legal literacy around workplace and academic rights, and adopt structural interventions that support survivors in navigating their disability rights within cancer recovery. By bridging this disconnect between survivorship and disability, future research, clinical practice, and policy can more effectively support and educate AYA survivors in participating in daily life, independent of the degree of lasting effects that persist in their post-treatment lives.

## Supplementary Information

Below is the link to the electronic supplementary material.

Supplementary file1 (DOCX 2.36 MB)

Supplementary file2 (DOCX 8 KB)

## Data Availability

No datasets were generated or analysed during the current study.
