# Supplementary material for: Disability experiences and quality of life in adolescent and young adult cancer survivors: a scoping review
Source: J Cancer Surviv. 2026 Jan 16;20(Suppl 2):2259–75. doi: 10.1007/s11764-025-01964-5 (PMC13744558; doi:10.1007/s11764-025-01964-5)
Supplement: Supplementary file 2 [file 11764_2025_1964_MOESM2_ESM.docx]

Search Strategy:

From 2010 - present

**PubMed:**

("cancer surviv*"[ti]) AND ("disab*"[tiab] OR "impair*"[tiab] OR "Americans with Disabilities Act"[tiab]) AND ("young"[tiab] OR "youth"[tiab] OR "adolescent"[tiab] OR "young adult"[tiab] OR "Adolescent and Young Adult"[tiab] OR "child*"[tiab] OR "teen*"[tiab] OR "AYA"[tiab] OR "pedi*"[tiab]) —> 262 Articles

**Web of Science:**

(TI=”cancer surviv*”) AND ((AB=”disab*”) OR (AB=”impair*”)) AND (AB="young" OR AB="adolescent" OR AB="young adult" OR AB="Adolescent and Young Adult" OR AB=”AYA” OR AB=”Pedi*” OR AB=”teen*”) AND PY=(2010-2025) AND LA=(English)

**Academic Search Complete:**

TI("cancer surviv*")

AND (TI("disab*" OR "impair*" OR "Americans with Disabilities Act")

OR AB("disab*" OR "impair*" OR "Americans with Disabilities Act"))

AND (TI("young" OR "youth" OR "adolescent" OR "young adult"

OR "Adolescent and Young Adult" OR "child*" OR "teen*" OR "AYA" OR "pedi*")

OR AB("young" OR "youth" OR "adolescent" OR "young adult"

OR "Adolescent and Young Adult" OR "child*" OR "teen*" OR "AYA" OR "pedi*"))

**APA PsycINFO:**

TI("cancer surviv*")

AND

(TI("disab*" OR "impair*" OR "Americans with Disabilities Act")

OR

AB("disab*" OR "impair*" OR "Americans with Disabilities Act"))

AND

(TI("young" OR "youth" OR "adolescent" OR "young adult"

OR "Adolescent and Young Adult" OR "child*" OR "teen*" OR "AYA" OR "pedi*")

OR

AB("young" OR "youth" OR "adolescent" OR "young adult"

OR "Adolescent and Young Adult" OR "child*" OR "teen*" OR "AYA" OR "pedi*"))

**MEDLINE (EBSCO host)**

TI("cancer surviv*")

AND

(TI("disab*" OR "impair*" OR "Americans with Disabilities Act")

OR

AB("disab*" OR "impair*" OR "Americans with Disabilities Act"))

AND

(TI("young" OR "youth" OR "adolescent" OR "young adult" OR "Adolescent and Young Adult"

OR "child*" OR "teen*" OR "AYA" OR "pedi*")

OR

AB("young" OR "youth" OR "adolescent" OR "young adult" OR "Adolescent and Young Adult"

OR "child*" OR "teen*" OR "AYA" OR "pedi*"))

ERIC (1 Result)

TI("cancer surviv*")

AND

(TI("disab*" OR "impair*" OR "Americans with Disabilities Act")

OR

AB("disab*" OR "impair*" OR "Americans with Disabilities Act"))

AND

(TI("student*" OR "school*" OR "adolescent" OR "child*" OR "teen*" OR "pedi*" OR "youth")

OR

AB("student*" OR "school*" OR "adolescent" OR "child*" OR "teen*" OR "pedi*" OR "youth"))

**SocINDEX with Full Text:**

TI("cancer surviv*")

AND

(

TI("disab*" OR "impair*" OR "Americans with Disabilities Act")

OR

AB("disab*" OR "impair*" OR "Americans with Disabilities Act")

)

AND

(

TI("young" OR "youth" OR "adolescent" OR "young adult" OR "Adolescent and Young Adult"

OR "child*" OR "teen*" OR "AYA" OR "pedi*")

OR

AB("young" OR "youth" OR "adolescent" OR "young adult" OR "Adolescent and Young Adult"

OR "child*" OR "teen*" OR "AYA" OR "pedi*")

**Health Source: Nursing/Academic Edition:**

TI("cancer surviv*")

AND

(

TI("disab*" OR "impair*" OR "Americans with Disabilities Act")

OR

AB("disab*" OR "impair*" OR "Americans with Disabilities Act")

)

AND

(

TI("young" OR "youth" OR "adolescent" OR "young adult" OR "Adolescent and Young Adult"

OR "child*" OR "teen*" OR "AYA" OR "pedi*")

OR

AB("young" OR "youth" OR "adolescent" OR "young adult" OR "Adolescent and Young Adult"

OR "child*" OR "teen*" OR "AYA" OR "pedi*")

)
